# A Cell-Based Approach to Study the Associations Between Mitochondrial Health, Early Life Exposures, and Consequent Health Outcomes

**DOI:** 10.3389/fpubh.2019.00036

**Published:** 2019-03-13

**Authors:** Dora Il'yasova, Alexander Kinev, Rose Grégoire, Craig C. Beeson

**Affiliations:** ^1^Department of Population Health Science, School of Public Health, Georgia State University, Atlanta, GA, United States; ^2^Creative Scientist, Inc., Durham, NC, United States; ^3^Department of Pharmaceutical and Biomedical Sciences, Medical University of South Carolina, Charleston, SC, United States

**Keywords:** biomarker, prenatal exposure, mitochondria, epidemiology, cell-based assay

Although humans, as placental mammals, possess a highly sophisticated system protecting the embryo/fetus *in uterus* from the environment, prenatal development might still be negatively affected via exposures to environmental factors (1–4). Many xenobiotic compounds in mothers' circulation (e.g., toxicants, drugs) can cross the placental barrier and reach developing tissues and organs, consequently altering cellular functions within the tissues. Among different cellular functions, mitochondrial metabolism is thought to be particularly sensitive to various stressors, including those produced by environmental toxicants (5, 6). Since mitochondria play a central role in cellular energy homeostasis and apoptotic/stress responses, it is not surprising that mitochondrial dysfunction is associated with numerous chronic diseases, such as neurodegenerative diseases, obesity, type-2 diabetes, along with a variety of cancers—the conditions which have been linked to environmental exposures (7–11). Many of the chronic disease conditions occurring later in life were proposed to originate from *in utero* exposures (1, 12). The proposed connections between *in utero* exposures to a wide variety of environmental toxicants and mitochondrial dysfunction have been documented by multiple animal studies. For example, maternal exposures to small-sized particulate matter and cigarette smoke were associated with a reduction in the mitochondrial (mt)DNA copy number per cell and a lower expression of oxidative phosphorylation proteins in the offspring (13). Exposure to cadmium, a toxic component of cigarette smoke, has been shown to inhibit mitochondrial respiration while simultaneously increasing reactive oxygen species (ROS) production (14). Prenatal exposure of rats to perfluorooctane sulfonate (PFOS) induced apoptosis in offspring heart tissue through the mitochondria-mediated apoptotic pathway (15). *In utero* exposure to arsenic impaired mitochondrial respiration with subsequent cardiac myopathies (16) and neurotoxicity (11). It was further shown that obesogens—chemicals that increase an offspring's weight—may also act as mitochondrial toxicants (10). Established obesogens, organotin compounds inhibit mitochondrial ATP production (17). Early-life exposure to the endocrine disruptor bisphenol A (BPA) has been linked with the development of obesity (18). At the same time, exposure to BPA has been associated with hypermethylation of the master mitochondrial regulatory gene PGC-1α and reduced mitochondrial respiration and ATP production (19). All these examples summarized in Table 1 corroborate the hypothesis that prenatal exposures to xenobiotics might influence post-natal development, with mitochondrial metabolism playing an important role in such a connection.

**Table 1:** Mitochondrial function impairment after prenatal exposure to environmental toxicants in animal studies.

**Toxicants**	**Effect**	**References**
Cigarette smoke and small-sized particulate matter	Reduction in the mitochondrial (mt)DNA copy number per cell and lower expression of oxidative phosphorylation proteins	(13)
Cadmium	Inhibition of mitochondrial respiration, increased ROS production	(14)
PFOS (perfluorooctane sulfonate)	Mitochondria-mediated apoptosis heart tissue	(15)
Arsenic	Impairment of mitochondrial respiration	(16)
Organotin compounds (obesogens)	Inhibition of mitochondrial ATP production	(17)
Bisphenol-A (BPA)	Reduced mitochondrial respiration and ATP production, hyper methylation of the master mitochondrial regulatory gene PGC-1α	(18, 19)

The idea of a prenatal origin of childhood obesity attracts attention, because lifestyle interventions implemented amongst this age group have not been effective in lowering adiposity among corpulent children (20). If prenatal environment plays an important role in inducing individual predispositions to excessive adiposity, then typical postnatal interventions may have little-to-no effect on obesity in both adults and children. Given the rapid increase in childhood obesity prevalence, evaluation of how prenatal xenobiotic exposures affect mitochondrial metabolism in embryo-fetal development have become an important focus of biomedical research. Some studies estimate that *in utero* exposure to BPA alone may contribute to 12,404 cases of childhood obesity, with a U.S. societal cost of $1.49 billion (21). Similarly, prenatal exposure to BPA in the European Union has a 20–60% probability of initiating 42,400 cases of childhood obesity (22). However, the confirmatory evidence from prospective human epidemiological studies that link prenatal exposure to altered mitochondrial function is currently lacking. In this opinion manuscript, we suggest that the assessment of mitochondrial function at birth in cells isolated from the umbilical cord blood might help to identify prenatal exposures through their alteration of mitochondrial bioenergetics in newborns, which can be subsequently linked to postnatal health outcomes. Next, we will discuss both the strengths and limitations of such an approach.

## Proposed Strengths

One of the important considerations in the human assessment of mitochondrial health is accessibility to a sufficient amount of biological material. This issue is especially pertinent to the development of biomarkers in the pediatric population and is even more challenging for epidemiological studies in human embryos, fetuses, or neonates (23). Umbilical cord blood can be obtained in an entirely non-invasive manner, and it represents a rich source of fetal cells—especially the highly proliferative stem and progenitor cells. Our previous work indicates that a population-wide collection of donor-specific endothelial colony-forming cells (or ECFC's) is possible to obtain because ECFCs can be isolated from cord blood with a high success rate (>90%) (24). ECFCs can be expanded *in vitro*, thereby providing sufficient amounts of biological material for multiple cell-based assays, including those for mitochondrial function (25). Moreover, a collection of donor-specific ECFCs can be viewed as a panel of tissues representing human neonatal population to enable assessment of individual variability in responses to mitochondrial toxicants.

In the past, mitochondrial health was assessed indirectly from changes in mtDNA and enzymatic activity related to different mitochondrial functions. Such measurements in peripheral blood mononuclear cells provided the first evidence that mitochondrial health can be associated with many chronic health conditions, including but not limited to diabetes, cardiovascular disease, and common neurodegenerative disorders (26–31). Donor-specific cells isolated from cord blood provide an opportunity to use the current high-resolution respirometry technology (32) to directly assess mitochondrial bioenergetic profiles within newborns. The sequential additions of different metabolic modulators, known commonly as a “stress test,” determines several parameters in one experiment: the glycolytic activity, the ATP-linked respiration, proton leak, basal and maximal respirations, and finally the non-mitochondrial respiration. Each of these parameters presents interpretable measurements that are the biomarkers of mitochondrial health (6). For example, when cells are placed in identical experimental conditions, lower ATP-linked respiration could indicate damage to substrate uptake or oxidative phosphorylation. A greater proton leak could indicate increased uncoupling protein (UCP) activity that has been related to the “less economical” bioenergetics with a higher intensity of fat oxidation and a lower predisposition to obesity and diabetes (32). Alternatively, a high proton leak may indicate a response to oxidative stress (32) or even damage to the inner mitochondrial membrane. Furthermore, the difference between the basal and maximal respiration presents the reserve bioenergetic capacity, a sensitive parameter correlated to cellular stress (6). Recently, a combination of these parameters were fused into a single Bioenergetic Health Index (BHI), which has been proposed to represent the donor's composite mitochondrial profile. Such an index could provide a dynamic indicator of bioenergetic health measured in platelets and leukocytes. Ultimately, the BHI has the potential to be a new biomarker in the assessment of patient health via presentation of both the prognostic and diagnostic values (6). However, even peripheral blood measurements that are considered minimally invasive can be invasive for newborns. Fortunately, using umbilical cord blood cells presents a non-invasive alternative. Thus, using cord blood cells as a platform to assess a newborn's mitochondrial health provides the direct measurements of mitochondrial bioenergetics in this otherwise difficult-to-study population.

## Unanswered Questions and Limitations

When assessing mitochondrial health, the major question is as follows: *which human cells should be used to assess mitochondrial function in an individual?* It has been argued that the mitochondrial respiration profile of circulating leukocytes and platelets can serve to assess mitochondrial function in other tissues, or more specifically, in that of a skeletal muscle (33, 34). Similarly, it is not certain whether the bioenergetic profile of umbilical cord blood cells correlates with cells comprising other fetal/newborn tissues. In humans, these correlations are practically impossible to assess due to ethical barriers; however, such research can be conducted in animal models.

Another question is whether a mitochondrial stress test profile has the ability to measure an individual characteristic that is stable over time. For epidemiological assessment, low within-person variability of a biomarker is especially important in evaluating its association with a disease of long latency (35). If within-person variability is large compared to the overall between-person variability, such a biomarker cannot represent an individual characteristic, differentiating one study participant from another. With respect to mitochondrial health, within-person variability can be assessed by comparing cells derived from the same donor vs. variability between cells derived from different donors using a mitochondrial stress test profiles, individual stress parameters and/or the composite BHI measurements. With respect to peripheral blood cells, within- and between-person variability can be assessed from blood specimens obtained from the same donors procured at different time points. One important caveat is that such an evaluation must be performed carefully, especially considering the circadian differences in circulating nutrients and/or physical activity that may influence the bioenergetic profiles of cells isolated from peripheral blood. Since peripheral blood cells cannot produce viable primary *in vitro* cell lines, the omission of the confounding effect of recent exposures would be problematic. In contrast, using primary cultures of cord blood progenitor cells could help to bypass the confounding influence of recent exposures, as these cells produce viable donor-specific cell lines and can be cultured in identical conditions. However, there is only one chance to obtain a cord blood specimen—during a delivery. How, then, will within-person variability of mitochondrial health can be evaluated in cord blood-derived cells? To bypass such a limitation, we propose to develop multiple cell lines from a single donor/newborn for the assessment of within-person variability.

Finally, feasibility considerations for large-scale epidemiological studies bring up a question of utilization of the existing cord blood biobanks. The current cell isolation methods do not consistently provide viable, proliferating cells from either frozen whole blood or partially purified blood samples. Therefore, fresh umbilical cord blood samples should be used to isolate cells, which then can be stored for a long period of time (several years in our experience) for cell-based assays. Thus, the need to use fresh cord blood specimens considerably limits the use of the existing children cohorts, for which the frozen cord-blood specimens are available.

## Conclusions

Mitochondrial health can be assessed at birth in cell-based assays using cells that are isolated from umbilical cord blood. Animal studies present enough evidence connecting prenatal exposure to environmental toxicants and mitochondrial health in different tissues of the offspring, demonstrating that such a connection might play an important role in human health. Animal studies can answer questions about correlations between mitochondrial health biomarkers obtained from cord-blood cells and other tissues in the offspring, ultimately proving or disproving that the assessment of mitochondrial health in one tissue can be extrapolated to the entire individual organism. However, only human studies can characterize within- and between-person variability of mitochondrial health biomarkers. With respect to the hypothesis of mitochondrial involvement in early origins of disease, using cord blood cells presents a unique opportunity for studying the connections between prenatal exposures, mitochondrial health, and long-term health outcomes.

## Further Readings

For further readings on the topic, we suggest the following references: (36–53).

## Author Contributions

DI and AK conceived the idea of this opinion and wrote the initial text. CB provided his expertise in mitochondrial science. RG helped to edit the initial text, researched the published literature for correct references, and wrote the part of the manuscript about animal models.

### Conflict of Interest Statement

AK is a founder and executive director of Creative Scientist, Inc. The remaining authors declare that the research was conducted in the absence of any commercial or financial relationships that could be construed as a potential conflict of interest.
